# The relationship between children’s rumination and parental rumination, worry and depressive symptoms

**DOI:** 10.1192/j.eurpsy.2024.739

**Published:** 2024-08-27

**Authors:** J. Szőke, M. Klein, Á. Örkényi, G. Kökönyei

**Affiliations:** ^1^ Institute of Psychology, ELTE Eötvös Loránd University; ^2^ Doctoral School of Psychology, ELTE Eötvös Loránd University; ^3^3NAP3.0-SE Neuropsychopharmacology Research Group, Hungarian Brain Research Program, Semmelweis University, Budapest, Hungary

## Abstract

**Introduction:**

Rumination is a transdiagnostic phenomenon that is linked to psychological and physical symptoms not only in adulthood but also in childhood. Several distal and proximal factors are believed to underlie the development of ruminative tendencies, with parental characteristics and modelling being among those with a potential association with the increased levels of children’s rumination.

**Objectives:**

The primary aim of the study was to investigate the link between rumination in children and parental functioning, including rumination, worry and depressive symptoms. Additionally, we aimed to test the association between rumination and psychological and somatic health in a sample of healthy children and early adolescents.

**Methods:**

153 children (87 girls, mean age = 10.74; SD = 0.91 years) and their parents (130 females, mean age = 42.65; SD = 4.08 years) participated in the study. For children, Kid Rumination Interview (KRI; Baiocco et al., 2017) was used, alongside the assessment of nine subjective health complaints. KRI employes 4 images to measure the frequency of rumination. Self-reported questionnaires were also completed by parents to report on worry, rumination, and depressive symptoms.

**Results:**

Contrary to our expectations, there was no significant association between children’s rumination and parental rumination (r = .06, p = .506), worry (r = -.02, p = .850) and depressive symptoms (r = -.01, p = .979). Psychosomatic complaints in children exhibited a positive albeit weak association with parental depressive symptoms (r = .17, p = .046). Regression analysis revealed that the frequency of rumination occurring in the four situations associated significantly with psychosomatic symptoms (β = .266; t = 3.321; p = .001) after controlling for sex and age.

**Conclusions:**

Our findings are in line with previous studies demonstrating the relationship between rumination and psychosomatic symptoms in older adolescent samples. However, parental perseverative cognitions and depression were unrelated to ruminative tendencies in children. Nevertheless, the modest sample size and the employment of a different assessment approach compared to self-report questionnaires may have influenced our findings.

**Disclosure of Interest:**

None Declared

